# The Changing Epidemiology of Murray Valley Encephalitis in Australia: The 2011 Outbreak and a Review of the Literature

**DOI:** 10.1371/journal.pntd.0002656

**Published:** 2014-01-23

**Authors:** Linda A. Selvey, Lynne Dailey, Michael Lindsay, Paul Armstrong, Sean Tobin, Ann P. Koehler, Peter G. Markey, David W. Smith

**Affiliations:** 1 School of Public Health, Curtin University, Perth, Western Australia, Australia; 2 Independent consultant, Perth, Western Australia, Australia; 3 Environmental Health Directorate, WA Health, Perth, Western Australia, Australia; 4 Communicable Disease Control Directorate, WA Health, Perth, Western Australia, Australia; 5 Communicable Diseases Branch, Health Protection NSW, NSW Health, Sydney, New South Wales, Australia; 6 Communicable Disease Control Branch, SA Department for Health and Ageing, Adelaide, South Australia, Australia; 7 Centre for Disease Control, Department of Health, Northern Territory, Australia; 8 School of Pathology and Laboratory Medicine, Faculty of Medicine, Dentistry and Health Sciences, University of Western Australia, Perth, Western Australia, Australia; U.S. Naval Medical Research Unit Six, United States of America

## Abstract

Murray Valley encephalitis virus (MVEV) is the most serious of the endemic arboviruses in Australia. It was responsible for six known large outbreaks of encephalitis in south-eastern Australia in the 1900s, with the last comprising 58 cases in 1974. Since then MVEV clinical cases have been largely confined to the western and central parts of northern Australia.

In 2011, high-level MVEV activity occurred in south-eastern Australia for the first time since 1974, accompanied by unusually heavy seasonal MVEV activity in northern Australia. This resulted in 17 confirmed cases of MVEV disease across Australia. Record wet season rainfall was recorded in many areas of Australia in the summer and autumn of 2011. This was associated with significant flooding and increased numbers of the mosquito vector and subsequent MVEV activity. This paper documents the outbreak and adds to our knowledge about disease outcomes, epidemiology of disease and the link between the MVEV activity and environmental factors.

Clinical and demographic information from the 17 reported cases was obtained. Cases or family members were interviewed about their activities and location during the incubation period.

In contrast to outbreaks prior to 2000, the majority of cases were non-Aboriginal adults, and almost half (40%) of the cases acquired MVEV outside their area of residence. All but two cases occurred in areas of known MVEV activity.

This outbreak continues to reflect a change in the demographic pattern of human cases of encephalitic MVEV over the last 20 years. In northern Australia, this is associated with the increasing numbers of non-Aboriginal workers and tourists living and travelling in endemic and epidemic areas, and also identifies an association with activities that lead to high mosquito exposure. This outbreak demonstrates that there is an ongoing risk of MVEV encephalitis to the heavily populated areas of south-eastern Australia.

## Author Summary

An outbreak of Murray Valley encephalitis with 17 confirmed cases occurred across Australia in 2011. This outbreak involved parts of Australia where cases had not occurred for many decades. The epidemiology in this outbreak reflects a change that has occurred over the past 15 years, with more non-Aboriginal cases, fewer children and more cases that were not resident where they acquired the infection than had been observed prior to 2000. The outbreak was associated with significant flooding in many parts of Australia and most cases reported either outdoor activities where mosquito exposure was highly likely or significant mosquito exposure.

## Introduction

Murray Valley encephalitis virus (MVEV) causes the most serious of the mosquito-borne virus diseases endemic to Australia. It also occurs on the island of New Guinea, and little is known about its epidemiology there [1]. It is a member of the Japanese encephalitis serogroup of flaviviruses and was responsible for four large outbreaks of encephalitis on the east coast of Australia in the early part to the 20th century (ranging from 21 to 114 cases) [2], and subsequently confirmed epidemics in 1951 (45 cases) and 1974 (58 cases) [1]. Since then the virus has been maintained in enzootic foci in the north of Western Australia (WA) and the Top End of the Northern Territory (NT), primarily in a cycle between water birds and *Culex annulirostris* [1], with possible contributions from other enzootic foci [3]. With the exception of one case in New South Wales (NSW) in 2008, encephalitis due to MVEV between 1975 and 2010 has been confined to these parts of Australia and adjacent areas further south in WA, in central Australia and in northern Queensland (Qld). Spread of MVEV outside these enzootic foci is thought to be due to rainfall and flooding that allowed movement of infected water birds to previously arid environments. Persistence in desiccation-resistant mosquito eggs may also contribute to outbreaks in previously arid areas, and the existence of cryptic enzootic foci has also been postulated [3]–[5].

The majority of infections with MVEV are asymptomatic or cause a non-specific febrile illness usually accompanied by headache, myalgia and occasionally rash [6]. However, in approximately 1∶150 to 1∶1000 infections with MVEV, clinical encephalitis results [6]. After the incubation period of up to 4 weeks, clinical cases usually present with fever (commonly accompanied by convulsions in children), headache, malaise, and altered mental status, which may be followed by progressive neurological deterioration, parkinsonian tremor, cranial nerve palsies, peripheral neuropathy, coma, flaccid paralysis, and death [6]. The reported case fatality rate of encephalitic MVEV is between 15–30%, with long-term neurological sequelae occurring in 30–50% of survivors and only around 40% recovering completely [6].

Sentinel chicken programs, where flocks of flavivirus-naïve chickens are kept specifically for regular testing for MVE infection, are in place in most parts of Australia where MVEV activity has occurred, with the role of providing an early warning system for MVEV activity [7]. The chickens are bled regularly for evidence of seroconversion to MVEV and other flaviviruses. Some states and territories also have mosquito trapping programs to monitor virus activity in mosquito populations, but difficulty of access and technical limitations currently prevent them being used for real-time monitoring [8].

As there are so few human cases of MVEV disease in Australia, there are limited data about the epidemiology and outcomes, and virtually no information about individual case risk factors.

In March to May of 2011, high level MVEV activity with human infections occurred in SE Australia for the first time since 1974, accompanied by an unusually heavy seasonal MVEV activity in the NT and northern and central WA [9].

During this period there was also a major national outbreak of encephalitis in horses, predominantly in NSW and Victoria. The majority of cases had laboratory evidence of flavivirus infection (either Kunjin or MVEV). This was the first such outbreak since 1974 [10].

In this report we document the human MVEV cases and add to our knowledge about disease outcomes, epidemiology of the disease, individual risk factors and the link between the heightened MVEV activity and environmental factors.

## Methods

The investigation and reporting of the MVEV cases was undertaken as part of normal Notifiable Diseases follow up by public health officials in state/territory health departments. As such, ethics committee approval for this study was not required.

MVEV disease is a ‘notifiable’ communciable disease in all Australian States and Territories, meaning that clinicians and laboratories are required by law to report cases to local health authorities. Cases of MVEV disease, either encephalitic or non-encephalitic, with a date of onset in 2011 were extracted from state and territory surveillance systems. Each case fulfilled the national case definition for a confirmed case of MVEV infection [11] requiring definitive laboratory evidence and clinical evidence. Clinical information was obtained on each case as part of normal case follow up. Additional clinical information for eight cases hospitalised in WA (seven infected in WA and one in NSW) was also obtained from a recent publication [12]. Cases or their family members were interviewed about their activities and their location during the incubation period (three weeks prior to illness onset). Activities that involved significant exposure to mosquitoes (eg outdoor activities, camping, fishing or others describing exposure to large numbers of mosquitoes) were classified as high-risk activities.

Details for an additional case in a tourist who fell ill after returning home and who was not notified in Australia, were accessed via ProMED mail [13].

Cases were classified as Aboriginal according to the National Health Data Dictionary (Aboriginal but not Torres Strait Islander origin) [14], and all others as non-Aboriginal. Cases who lived in the area in which they acquired MVEV infection were classified as resident. Other cases, who were either travelling in or working temporarily in the area in which they acquired MVEV infection, were classified as non-resident.

Details of state and territory sentinel chicken programs for MVEV surveillance and mosquito trapping have been described elsewhere [9], [15].

## Results

### Human cases of MVE

**Figure 1:**
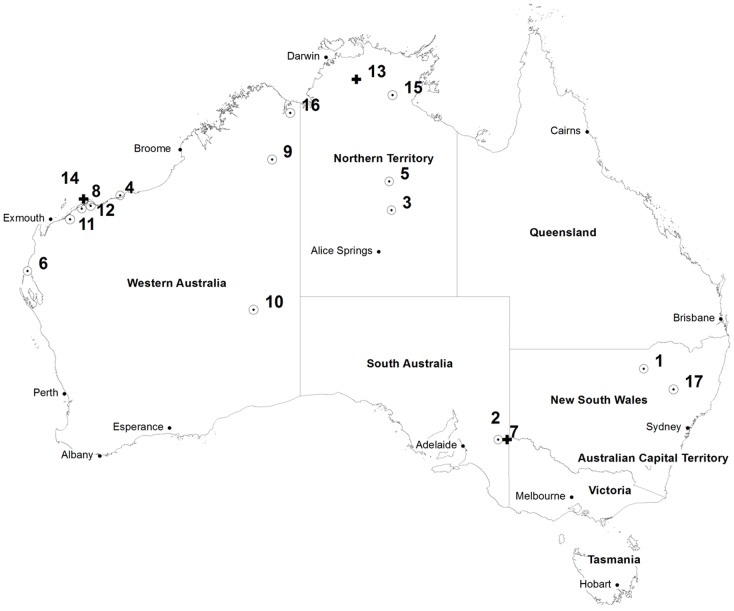
Geographical distribution of MVEV cases in Australia 2011. Legend: Each circle with a dot inside represents a case who survived; Each black cross represents a case who died. Note, cases 11 and 12; 2 and 7; and 8 and 14 occurred in the same location, and the symbols are shown side by side.

A total of 17 cases of MVEV disease were reported in 2011, including one case who acquired her infection in Australia but became symptomatic in Canada. Of these, nine cases (one death) were infected in WA, four cases (one death) in the NT, two cases (one death) in SA, and two cases in NSW. There were no cases notified in Qld, Tasmania or Victoria in 2011. All deaths were a direct result of encephalitis. All but one case had a date of onset between March and May 2011, and the final case occurred in December 2011 in NSW at the beginning of the next summer. See Table 1 and Figure 1 for further details.

**Table 1:** Cases of clinical MVEV in Australia, 2011.

Case	Date of onset	Infection region	State	Age	Sex	Ethnicity	Encephalitis	Exposure and risk	Residential status	Last follow up	Outcome
○1	03/03/2011	North-western NSW	NSW	63	F	NA	Encephalitis, full recovery	High risk: rural resident, flooding, high numbers of mosquitoes noted.	Resident	On discharge from hospital	Recovered [12]
○2	12/03/2011	Riverland	SA	47	M	NA	Encephalitis, unspecified residual neurological deficit	High risk: outdoor activities. Extensive travel in the Murray River valley in Victoria and/or NSW adjacent to SA's Riverland region. Noted multiple mosquito bites	Resident*	Unknown	Neurological deficit, improvement expected.
○3	20/03/2011	Southern Barkly Region	NT	1	M	A	Encephalitis, moderate neurological deficit	Was in an area with high rates of MVEV activity, but no specific mosquito exposure reported.	Resident	Unknown	Epilepsy and developmental delay.
○4	21/03/2011	Pilbara	WA	50	F	NA	Non-encephalitic	High risk: multiple bites confirmed – most evenings	Resident	Unknown	Persistent headache.
○5	21/03/2011	Barkly	NT	33	M	NA	Encephalitis, global neurological deficit	Was in an area with high rates of MVEV activity, but no specific mosquito exposure reported.	Resident	Eight months after onset	Gradual recovery over 4 months, with minor residual memory impairment and irritability.
○6	22/03/2011	Midwest	WA	41	F	NA	Encephalitis, moderate neurological deficit	High risk: night fishing	Resident	Post-discharge, time undetermined	Impaired cognition, hypertonia [12]
○7	23/03/2011	Riverland	SA	27	M	NA	Encephalitis, fatal	High risk: extensive outdoor activities and travel in the Murray River valley in Victoria and/or NSW adjacent to SA's Riverland region.	Resident*	60 days after onset	Died
○8	30/03/2011	Pilbara	WA	61	M	NA	Encephalitis, fatal	High risk: camping	Non-resident (employment)	18 days after onset	Died
○9	10/04/2011	Kimberley	WA	29	M	NA	Encephalitis, severe residual neurological deficit	Was in an area with high rates of MVEV activity, but no specific mosquito exposure reported.	Non-resident (employment)	8 months after onset	Wheelchair-bound, mixed flaccid paralysis and hypertonicity of limbs [12]
○10	19/04/2011	Goldfields	WA	25	M	A	Encephalitis, full recovery	High risk: spent evenings outdoors with stagnant water post flooding and noted large numbers of mosquitoes	Resident	At discharge from hospital	Full recovery [12]
○11	22/04/2011	Pilbara	WA	25	M	NA	Encephalitis, moderate residual neurological deficit	High risk: camping beside river	Resident	At discharge from hospital	Impaired cognition, gait disturbance, severe memory impairment, dysphagia [12]
○12	4/05/2011	Pilbara	WA	67	F	NA	Encephalitis, mild residua	High risk: mosquito bites confirmed; camping and noted high levels of mosquitoes.	Non-resident (tourist)	At discharge from hospital	Impaired cognition, mild dysphagia [12]
○13	14/05/2011	Top End	NT**	19	F	NA	Encephalitis, fatal	High risk: camping	Non-resident (tourist)	10 days after onset	Died
○14	15/05/2011	Pilbara	WA	2	F	NA	Encephalitis, full recovery	High risk: many confirmed mosquito bites in week preceding illness.	Resident	At discharge	Recovered [12]
○15	25/05/2011	Arnhem Land	NT***	63	F	NA	Encephalitis, confusion	High risk: caravan and regional travel.	Non-resident (tourist)	One month after onset	Recovered
○16	May 2011	Kimberley	WA	2	F	A	Encephalitis, unspecified neurological deficit	Was in an area with high rates of MVEV activity, but no specific mosquito exposure reported.	Non-resident (tourist)	Unknown	Neurological deficit.
○17	06/12/2011	North-western NSW	NSW	25	F	NA	Encephalitis, unspecified neurological deficit	High risk: rural outdoor employment. High numbers of mosquitoes noted. No repellent used.	Non-resident (employment)	Six weeks after onset	No gross neurological deficits. Continuing headaches and fatigue

KEY: State: NSW – New South Wales, NT – Northern Territory, SA – South Australia, WA – Western Australia; Gender, M – male, F – female; Ethnicity, NA – non-Aboriginal, A – Aboriginal.

Resident in an area of MVEV activity, but had also travelled into adjacent areas with MVEV activity. **Overseas visitor. ***NSW resident.

Fourteen cases were adults, and three cases were children aged two years and under. Nine of the cases were female and eight were male. Of the adult cases, the median age was 37, ranging from 19–67 years. Fourteen of the 17 cases were non-Aboriginal people. Ten of the cases were residents of the area where they presumably acquired MVEV, the remainder were non-residents; either tourists or people temporarily employed in the MVEV regions.

Three cases died, giving a crude case fatality rate of 18% amongst confirmed cases. Of the 14 survivors, four made a full recovery. Of the remaining ten, eight cases had neurological deficits, (two of which were mild, five severe and one unspecified) and two cases reported persisting headaches and/or fatigue.

All cases occurred in areas known to have had clinical MVEV cases or sentinel chicken seroconversions to MVEV since 1974. In 2011 evidence of MVEV activity in sentinel chickens and/or horses was recorded in all areas that had clinical cases.

Thirteen cases (77%) reported outdoor activities that posed a high risk of mosquito exposure. At the time of diagnosis, four cases had physical evidence of recent mosquito bites, and a further three cases had been in situations where they had observed high levels of mosquitoes during periods of known MVEV activity. In addition, two other cases resided in areas where extensive flooding had occurred prior to the likely time of infection.

### Environmental conditions

Much of Australia experienced a very wet 2010/11 summer and autumn with all States and Territories recording above-average rainfall [16], [17]. Areas of very-much-above-average rainfall were widespread across Australia; with the exception of the southwest of WA [16]. Much of Victoria, southern NSW, eastern SA, and parts of WA, NT and Qld, had falls that ranked as the highest on record [16]. The wet conditions during summer resulted in major flooding in many areas across the country including Qld, parts of NSW, Victoria and northern Tasmania, and the western part of the Midwest region of WA [16]. This flooding extended to large wetland systems throughout the Murray-Darling Basin and the Lake Eyre Basin, where the highest numbers of waterbirds were recorded since 1984 [18]. NSW had further heavy rainfall in November, 2011, with associated flooding in a number of areas including in proximity to the location of the December case [19].

High numbers of mosquitoes were trapped in inland NSW, with 102 arbovirus isolates, but no MVEV was isolated [9].

### Sentinel chicken surveillance

Sentinel chicken surveillance programs were in place in WA, NSW, inland Victoria and the NT during the 2010/11 season. The sizes of the sentinel flocks and locations and periods when seroconversions were recorded in 2011, are shown in Table 2. Other details of the programs, including testing methods are described elsewhere [9], [20].

**Table 2:** Sentinel chicken surveillance results in Australia, 2011.

State	Number of flocks	Number of chickens per flock	Total MVEV seroconversions	Date of first seroconversion	Date of last seroconversion
NSW	11	15 chickens/flock	10	21 Feb 2011	23 Mar 2011
			14	4 Dec 2011	13 Dec 2011
VIC*	13	20 chickens/flock	90	7 Feb 2011	9 May 2011
NT	10	10 chickens/flock	15	14 Dec 2010	2 Jun 2011
WA	30	12 chickens/flock	219	Feb 2011	Jun 2011

Results include a combination of seroconversion to flavivirus only and to MVE specifically.

Activity of MVE during the 2011 season was first detected in sentinel chicken flocks in the NT in December 2010 and in WA, Victoria and NSW in February 2011 (Table 2) [21], [22]. In March, substantial seroconversions were detected across most of northern and central WA [21], NSW [22], and in the Central Australian region of the NT. MVEV activity continued through April, and in May, MVEV was detected in WA sentinel chickens located as far south as latitude 29°S. MVEV activity was first detected at this site in 2000 and had not been found that far south since then [21]. Coinciding with the human case in NSW in December 2011, MVEV seroconversions occurred over a limited period in early December in the same region [22]. Since 1974, sentinel chicken seroconversions had occurred in inland NSW in three seasons between 2000 and 2010, and in SA (not in a formal sentinel chicken surveillance program) and Victoria in 2008 [2].

### Public health response

Each State and Territory has its own public health response to MVE activity, with actions taken in response to epidemiological analysis of vector numbers, rainfall, historical risk periods, sentinel chicken results or human cases.

Victoria implemented mosquito control programs, pumping of flood waters, health alerts to the health system and testing for MVEV. Television, radio and print notices were utilised advising personal protection and mosquito avoidance in conjunction with Tourism Victoria [23].

Western Australia issued four statewide media statements in 2011. These followed the detection of MVE in chickens in the Kimberley (February), widespread activity across the state (March), a human case in Carnarvon (April), and new detections of MVEV antibodies in sentinel chickens in the Midwest/Wheatbelt and Goldfield regions (May). The statewide media statements were further publicised at a regional and local level by population health units and local governments, and included pictorial warnings distributed to at-risk small communities. Local government mosquito management programs were also escalated in response to detection of seroconversions in sentinel chickens. Every wet season, Western Australia also routinely issues an alert message on a radio station specifically aimed at tourists, which is upgraded following seroconversion of sentinel chickens.

SA issued media releases after the first case was notified, as well as providing detailed clinical information to doctors and diagnostic laboratories. Mosquito monitoring and control activities were enhanced and mosquito avoidance health promotion activities were increased.

NSW issued a statewide media statement in February 2011 following the first MVEV seroconversions in sentinel chickens. A NSW mosquito control expert panel was convened in March 2011 following the detection of the first human case of MVEV, additional sentinel flock seroconversions and requests to provide advice on mosquito control in flood-affected areas. Four more statewide media statements were issued in March and April 2011, together with alerts to clinicians and laboratories. Letters were sent to local councils with advice from the expert panel on mosquito control measures and other risk reduction measures, and with recommendations to promote risk prevention through communications and posters. Regional public health units in affected areas also conducted targeted public communications. NSW issued additional statewide media statements in December 2011 and January 2012 following the detection of additional sentinel flock seroconversions and the detection of the second human case of MVEV.

In the Northern Territory, a health warning in the form of a media release was issued at the start of the high risk period for MVE. A heightened media alert was issued and health care providers were advised after the sentinel chicken seroconversions. Each case was also followed up with a media release.

## Discussion

2011 saw a dramatic increase in MVEV activity in endemic regions, and the re-emergence of MVEV in south-eastern Australia [6]. There were 17 cases recorded across WA, SA, NSW and NT, which is the largest number of cases since 1974 and the first large multi-state outbreak since 2000, when there were fourteen cases across central and northern WA, NT and northern SA [7].

The MVEV activity and the resulting human infections arose from two separate but overlapping sets of environmental conditions. The virus activity and human cases in WA and the NT were a result of heavy seasonal rainfall in the northern and central areas of these two jurisdictions. Activity in southeastern Australia followed the extensive rainfall and flooding in the Murray-Darling basin and adjacent areas in Queensland, NSW, Victoria and SA.

The case-fatality rate in this outbreak was 18%, which is similar to the 20% case-fatality rate during the last major outbreak of MVE in 1974 [6]. This likely reflects the lack of advancements in specific treatments for MVE over the past four decades beyond supportive therapy in intensive care, which has been available in Australia for some time.

The cases in 2011 followed the usual seasonal patterns for MVEV, with sixteen of the cases occurring in autumn (March–May) with no cases during winter or spring and a single case in northern NSW in the following summer during a new period of heavy rainfall flooding and MVEV activity in the sentinel chickens.

**Figure 2:**
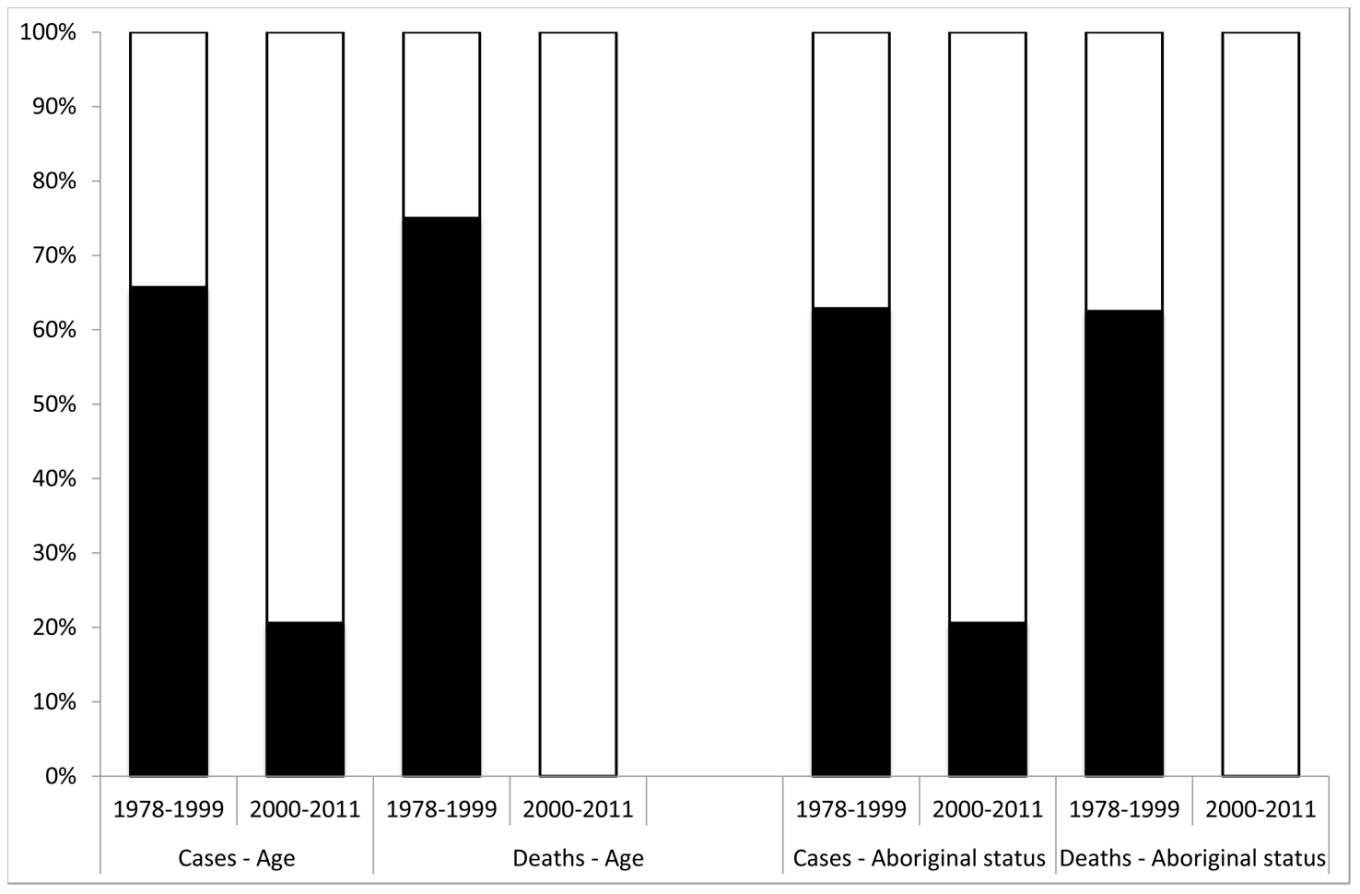
Age distribution and Aboriginal status of MVEV cases and deaths 1978–2011. Comparison of the distribution of age and Aboriginal status of MVEV cases between 1978–1999 and 2000–2011. Legend: The white unfilled areas in the graph represent either Adult or Non-Aboriginal cases. The black filled areas in the graph represent either Child or Aboriginal cases.

Of the 17 cases, 14 were non-Aboriginal, 14 were adults and almost half of the cases did not reside in the regions where they acquired MVE. This outbreak continues to reflect a change in the demographic pattern of human cases of encephalitic MVEV over the last 15 years from predominantly Aboriginal to predominantly non-Aboriginal and from paediatric to adult disease. This demographic shift was first noted in the 2000 outbreak that included nine cases in WA and five cases in the NT [7]. Early cases in south eastern Australia in 1951 were mainly children (25/40, 62%) [24], while in 1974 children comprised only 32% (7/22) [25]. No information was available about Aboriginality for those outbreaks. Of the cases that occurred between 1975 and 1999 [26]–[28] 23/35 (66%) were children (Figure 2) and 22 (63%) were Aboriginal, compared with 7/34 (21%) and 7/34 (21%) respectively for cases from 2000 to 2011 [12], [29]–[31]. A similar change has occurred in the age distribution of fatal cases (Figure 2). This may reflect, in WA and the NT in particular, increasing numbers of non-Aboriginal workers and tourists living and travelling in endemic and epidemic areas [32]–[34]. Serological surveys in the Kimberley region of Western Australia and in the Northern Territory showed increasing levels of immunity to MVE with age in Aboriginal communities in MVE-endemic areas, meaning that children are more susceptible than adults in these communities [28], [35]. As more non-immune people move into endemic and epidemic regions, either temporarily or permanently, it is perhaps not unexpected that a higher proportion of MVE cases would be non-Aboriginal adults.

In both the 2011 and 2000 MVE outbreaks, evidence of MVEV activity was recorded in areas where it has only rarely been recorded previously [29], [36]. In WA where the majority of cases were acquired, the areas of acquisition were predominantly south of the Kimberley region, whereas prior to 2000, cases more usually occurred in the Kimberley [37]. This change is likely to have been a major contributor to the shift in the epidemiology of the disease, as these more southerly areas have larger populations with a lower proportion of Indigenous people, mines and mining townships with predominantly temporary or itinerant populations and large amounts of commercial traffic between these areas and metropolitan Perth.

The majority of cases were involved in activities that lead to a high likelihood of exposure to mosquitoes. For example, cases reported fishing at dusk, camping near rivers and creeks and attending outdoor evening sports, which are all likely to increase mosquito exposure. Little is known about the predictors of clinically apparent MVEV infection in humans but while there may be a range of factors, animal studies with MVEV [38] and with other flaviviruses including Japanese encephalitis virus [39], [40] have demonstrated the importance of viral load. Mosquito saliva potentiates West Nile virus infection in mice, and this is thought to be related to local immunomodulation [41]. This suggests another potential mechanism by which multiple mosquito bites could facilitate clinical MVEV disease and emphasises that any reduction of mosquito biting is potentially beneficial.

Currently public health warnings advise all people in areas of MVEV activity to avoid all mosquito exposure. In 2011 these had been issued to residents living in areas of MVE activity before human cases were reported [42], [43]. However, without a formal evaluation, it is not possible to know whether these activities influenced behaviours or prevented additional MVE infections. Refining the warnings to target very high mosquito exposure activities may increase their effectiveness and credibility. Evaluation of the current public health measures is recommended, as well as consideration of additional social marketing activities should high-risk climatic conditions reoccur. Given the small numbers of cases, a MVEV-specific vaccine is unlikely to become available, but a new human chimeric JE vaccine has shown cross-protection against MVEV in mice [44].

Record wet season rainfall was recorded in many areas of northern Australia, central Australia and south-eastern Australia in the summer and autumn of 2011 [16], [17]. This was associated with significant flooding and increased numbers of the mosquito vector, *Culex annulirostris*, and subsequent MVEV activity [6], including widespread seroconversion of sentinel chickens [21], [22], [42]. In WA, the level of MVEV activity in sentinel chickens was one of the highest on record and was similar to that in 2000, when the last large outbreak of MVEV disease occurred in that state.

Sentinel chicken seroconversions in Central Australia are relatively rare and are usually associated with southern extension of the annual northwest monsoon activity [42], which may blow infected mosquitoes into the Barkly or Central Australian regions, and enable local amplification due to increased vector numbers associated with flooding [42]. Alternatively, the increase in southern rainfall and subsequent flowing of inland rivers might result in the southern migration of water birds which, combined with the local increase in vector numbers, could lead to MVEV activity in these areas. While the majority of sentinel chicken seroconversions occurred in areas of previously described MVEV activity, MVEV seroconversions in the Alice Springs area (Central Australia) had not been recorded since 2002 when a major local mosquito breeding site was drained [45]. However, the recent seroconversion to MVEV indicates that the local ecology in Alice Springs can still sustain these arboviruses given suitable climatic conditions [42].

In spite of widespread sentinel chicken activity and disease in horses in Victoria, encompassing an area where an estimated population of 270,000 people live, there was only one unconfirmed case of MVE [6], compared to much larger numbers in very sparsely-populated areas of WA and the NT in the same time-period. This was consistent with previous experience in these areas. A serosurvey conducted in Victoria following the 2011 outbreak found very low levels of MVEV antibody, especially in people born since the last epidemic there in 1974 [46]. This is a similar outcome to a serosurvey of people living in high-risk areas of Victoria and NSW conducted in 1991 [47]. These serosurveys suggest that the differences may be due to relatively less human infection in Victoria, possibly due to more effective mosquito control and/or less frequent high mosquito exposure activities in the urban areas of Victoria compared with large remote areas in northern Australia. The reduction in waterbird numbers in the Murray Darling Basin over time, as a result of increased utilisation of water for irrigation and clearing of wetlands may also have reduced the likelihood of mosquitoes being infected with MVEV [5]. The reduced density of waterbirds might explain the fewer MVEV case numbers in South-eastern Australia in 2011 compared to 1974, in spite of there being similar climatic conditions.

This outbreak shows that extensive MVEV activity continues to occur across Australia if the climatic conditions are suitable. The changing demography of human cases, together with the southward spread of MVEV activity in Western Australia shows that changing patterns of human movement and settlement and changes in ecological factors are continuing to influence the epidemiology of MVEV encephalitis in Australia. Continued MVEV surveillance and evidence-based public health responses are warranted given the serious consequences of MVEV encephalitis.
